# Supplementary material for: Chromothripsis during telomere crisis is independent of NHEJ, and consistent with a replicative origin
Source: Genome Res. 2019 May;29(5):737–49. doi: 10.1101/gr.240705.118 (PMC6499312; doi:10.1101/gr.240705.118)
Supplement: Supplemental Material [file supp_gr.240705.118_Supplemental_file_1.zip › contigs/annotated_contigs/DB115/contig.3.DB115_length_889_mean_cov_13.7457817773.docx]

**DB115_length_889_mean_cov_13.7457817773**

```
GGTTTCGTGGAGCAAGTACCACAGGGAAGGGGGGGGGAAGTGTGGGAAGTGGGTT|GGGGGGGGGGGGGGGGTGGGGAGGGGTGG|TGG
                                                        >chrX:44141866-44141895 + E=1e-
GGGGGGGGGGGGGGGGGGGGGGG|GTGTGTGTGTGTGTGTGTGTGTGTGTGTGTGTGTGTGTGTGTTTAATGTACCTAATTCTGTAAAG
02 p=0e+00              >chr6:155115922-155116466 + E=0e+00
GATTTTAAGTGATTTTTTCAAAGTGGACACAATAAAATAAAAACAATATGCGTGCATGTGTTTTATAAAAGTAATAAAACAAGTTATTC

TGCTTTTTCTAGTCTTAGTTACTTACTTACATATTTATTTGGGGGGTGTGATGTTCTTTTTAAAAGGAACTCCTGTGACACCTGTTACT

CCGGCCAATGTGGTCCAAGGCTTACCTGATCCGTGGGTATCTCAGATAACAAATACAGATACACTTGCGGCTGTAGCTCAGATCTTGCA

AAGTCCTCAAGGCCAGCAGGTGAGCGGTTTTTCTGTTATATCAAGAATAATTTAGAATTTAATATTTTCCTAATGTATTACTTTGTAAC

ATACCACCTAGAGAATATGTGCTTTTTTTAGTTTTATAAAAATGGGACCATTATGTATACTTTGTTACTTACTTAAGTCATTTAATAGT

GCATCATGAATGCTTTCATGTCATTAAAAATT|AC|ATCATAGAGCTACCAATAACCTCACTGTTTCTCCTCGCCAAGGAAGACAAGCT
                                 >chr6:154236259-154236496 + E=1e-130
GTTGTGCTTTATTTCAGTGTAAATCACCAGGTCAGAAATCCATATGTACACATGTTATCATCCAGTCCACCTAAATGTCTGGTTTTTGT

TCTAACGCCTGTACTTTTCTTGAACAATTCATCCTTGCTCTTATTTTGTTGGTTTTGTTTTCCCCACACCAGGAATTTCAGTGTCAGTG

TATT
```
